# Streptozotocin-induced diabetes disrupts the body temperature daily rhythm in rats

**DOI:** 10.1186/s13098-015-0035-2

**Published:** 2015-04-29

**Authors:** Angela M Ramos-Lobo, Daniella C Buonfiglio, José Cipolla-Neto

**Affiliations:** Department of Physiology and Biophysics, Institute of Biomedical Sciences, University of São Paulo, Av. Lineu Prestes, 1524, São Paulo, SP 05508-000 Brazil

**Keywords:** Daily rhythms, Type 1 diabetes mellitus, Insulin, Melatonin

## Abstract

**Background:**

In mammals, the temperature rhythm is regulated by the circadian pacemaker located in the suprachiasmatic nuclei, and is considered a “marker rhythm”. Melatonin, the pineal gland hormone, is a major regulator of the endogenous rhythms including body temperature. Its production is influenced by many factors, such as type 1 *diabetes mellitus*. In rats, diabetes leads to hypothermia and reduced melatonin synthesis; insulin treatment reestablishes both.

**Aim:**

To study the body temperature daily rhythm of diabetic animals and the effects of insulin and/or melatonin treatment on its structure.

**Methods:**

We studied the effects of streptozotocin-induced diabetes (60 mg/kg) on the body temperature rhythm of Wistar rats and the possible modifications resulting from early and late treatments with insulin (6U/day) and/or melatonin (daily 0.5 mg/kg). We monitored the daily body temperature rhythm, its rhythmic parameters (MESOR, amplitude and acrophase), glycemia and body weight for 55 days. Data were classified by groups and expressed as mean ± SEM. One-way ANOVA analysis was performed followed by Bonferroni posttest. Statistical significance was set at p < 0.05.

**Results:**

Diabetes led to complete disruption of the temperature rhythm and hypothermia, which were accentuated over time. All early treatments (insulin or/and melatonin) prevented the temperature rhythm disruption and hypothermia. Insulin plus melatonin restored the body temperature rhythm whereas insulin alone resulted less efficient; melatonin alone did not restore any of the parameters studied; however, when supplemented close to diabetes onset, it maintained the temperature rhythmicity. All these corrective effects of the early treatments were dependent on the continuous maintenance of the treatment.

**Conclusions:**

Taken together, our findings show the disruption of the body temperature daily rhythm, a new consequence of insulin-dependent diabetes, as well as the beneficial effect of the complementary action of melatonin and insulin restoring the normal rhythmicity.

**Electronic supplementary material:**

The online version of this article (doi:10.1186/s13098-015-0035-2) contains supplementary material, which is available to authorized users.

## Background

Circadian rhythms play a central role in both mental and physical health [1]. As all the circadian rhythms, body temperature is under the control of the circadian oscillatory system. Due to its robustness and the relative continuous monitoring easiness, body temperature has been established as a “marker rhythm” of the circadian pacemaker [2-4]. Scheer et al., described a dual effect of the suprachiasmatic nuclei (SCN) in thermoregulation in rats, i.e., daily synchronization and mediating the masking effect of light [5]. Besides that, the SCN are fundamental for the circadian timing of metabolic rhythms such as plasma glucose changes, cortisol and melatonin levels [6-11].

Evidence in literature shows a strong connection between circadian disruption and metabolic pathologies, and that the relationship is bidirectional [12,13]. Previous studies show that both experimental animals as well as patients with metabolic pathologies, such as obesity and type 2 diabetes mellitus also present circadian abnormalities [14,15]. On the other hand, mice with disrupted clock function develop metabolic pathologies, such as diabetes and obesity [16,17]. In spite of being a well-structured rhythm, body temperature is susceptible to chronic shifts in mealtime resulting in pathological changes of carbohydrate and lipid metabolisms daily rhythms [18]. These effects could be due to a misalignment of the body temperature, melatonin, and sleeping rhythms [19].

Melatonin is the main hormone produced by the pineal gland in mammals. Its synthesis is high during the night and low during the day, signaling to the body the daily and seasonal environmental photoperiod. It is known that melatonin synchronizes the body temperature rhythm in humans setting up the acrophases to the same hour every day [20]. Several studies show the importance of melatonin in the regulation of energy metabolism. The decrease or absence of endogenous melatonin secretion in rats may alter energy metabolism, resulting in increased body weight and visceral adiposity associated with glucose intolerance, insulin resistance, diabetes, dyslipidemia, and cardiovascular disease; melatonin administration in those animals is capable of reverting all the effects mentioned above [7,21-25].

It is not clear yet how melatonin modulates directly the body temperature rhythm; melatonin has a peripheral vasodilator effect and thus increases heat loss and decreases temperature in the dark phase in humans [26], and given its close relationship with energy metabolism, it might also be involved in body temperature regulation. It is known that body temperature is altered in metabolic pathologies, especially in diabetes mellitus. Type 1 diabetes mellitus (T1DM) is an autoimmune disease leading to the destruction of the insulin-producing pancreatic beta cells in the islets of Langerhans. This alteration can lead to serious diseases affecting the heart and blood vessels, eyes, kidneys, and nerves, together with a higher risk of developing infections. The disease can affect people of any age, but is most commonly diagnosed in children and young adults, and by the time of diagnosis, patients have very little endogenous insulin production; every year 78,000 children develop type-1 diabetes worldwide (IDF, 2011, [27,28]). Data in literature show that experimental diabetes, induced by streptozotocin (STZ) [29] or alloxan [30] reduces 24 h body temperature, leading to hypothermia and intolerance to cold temperatures [31]. Insulin treatment restores normal temperature [30,32,33]. This effect could be explained by the decreased brown adipose tissue thermogenesis, decreased shivering activity and lack of glucose uptake by muscle and adipose tissues, characteristic of diabetes [34-36]. When addressing melatonin synthesis in STZ-induced diabetic rats data in literature are discrepant. In spite of some contradictory evidence in the literature [37], data from our group, show a strong reduction (up to 50%) in melatonin synthesis in the pineal gland [38] and retina [39] of diabetic rats, and this effect is reversed by exogenous insulin. Moreover, in human T1DM there is a strong negative correlation between hyperglycemia and 6-sulphatoximelatonin production [38]. In any case, these findings indicate that melatonin plays an important role in metabolic alterations, especially diabetes mellitus [40].

The effect of insulin-dependent diabetes on the circadian profile of body temperature (BT) is still unknown. Giving that physiological temperature rhythm is essential to maintain normal body functions and that STZ-induced diabetic rats show a significant loss of endogenous melatonin; and considering the tight relationship between melatonin, insulin and metabolism, it is highly important to study the effect of this pathological state on the body temperature daily rhythm [40].

In the present study, we aimed to describe the daily rhythm of BT in STZ-induced diabetic animals through telemetric analysis. We also aimed to study the potential effects of insulin treatment, melatonin supplementation or a combination of both hormones on the rhythmic structure of temperature in diabetic animals.

## Material and methods

### Ethic statement

The Committee of Ethics in Animal Experimentation of the Institute of Biomedical Sciences, University of São Paulo, granted ethics approval for this study. All animal procedures were approved by the Ethics Committee on the Use of Animals of the Institute of Biomedical Sciences at the University of São Paulo, and were performed according to the ethical guidelines adopted by the Brazilian College of Animal Experimentation.

### Animal housing

Male Wistar rats (250 g) were obtained from the Institute of Biomedical Sciences, University of São Paulo, São Paulo, Brazil. The animals were kept under a constant 12 h: 12 h light–dark cycle (lights on at 7 AM; Zeitgeber Time [ZT] 0), in a temperature-controlled room (21 ± 2°C), with food and water ad libitum and housed in individual cages equipped with body temperature and locomotor activity recording.

### Surgical procedure and data acquisition

Body temperature recordings were obtained by telemetry (Mini Mitter, Bend, OR, USA). A small transponder (ER400 E-Mitter® Respironics – Mini Mitter, Bend, OR, USA) was implanted in the abdominal cavity of each rat. Briefly, animals were anaesthetized with a ketamine/xylazine solution (3:1). The ventral surface of the abdomen was shaved and a 2 cm incision was performed along the *linea abla* 1 cm below the diaphragm. The body of the E-Mitter was slipped into the abdominal cavity along the sagittal plane and dorsal to the digestive organs, then the abdominal cavity was massaged gently to allow the internal organs to settle. The animals were allowed 7 days to recover following surgery [41] and all efforts were made to minimize suffering. The data acquisition was performed by the software VitalView™ recording every 30 seconds during 55 days. Animal’s body weight and glycemia were measured weekly.

### STZ-induced diabetes, insulin and melatonin treatment

Diabetes was induced by a single intraperitoneal injection of STZ (60 mg/kg body weight; Sigma- Aldrich, St. Louis, MO) freshly diluted in citrate buffer (10 mM, Na citrate; pH 4.5). Tail blood was collected for glucose levels determination using a glucometer (Optium Xceed; Medisense, Abingdon, UK) 24 hours after the induction. Animals with glycemia > 200 mg/dL were considered diabetic. Insulin treated diabetic animals received 2 U subcutaneous long-action insulin (Glargina/Lantus; Sanofi-Aventis, Paris, France) at sunrise and 2 U regular insulin (Humulin R; Eli Lilly, Paris, France) plus 2 U long-acting insulin (Glargina/Lantus; Sanofi- Aventis) at sunset [39]. Melatonin was administrated in drinking water (0.5 mg/mL; pure melatonin was first dissolved in ethanol and added to distilled water to achieve the melatonin solution) only in the dark phase for the time determined in the experimental design.

### Analysis of rhythmic parameters

Temporal series were obtained with VitalView software (Mini Mitter, Bend, OR, USA) and individual thermograms were obtained from raw data using the El Temps® software (Diez-Noguera, Barcelona, Spain). For rhythmic analyses we used the Cosinor method using the COSANA software (Benedito Silva, São Paulo, Brazil). To generate the daily pattern, raw data was averaged in 1 h bins and analyzed as 24 h bins. The Cosinor method allows the daily rhythm of body temperature to be described in a simple cosine wave, which is typically characterized in terms of acrophase (measure of the crest time of a rhythm from the cosine wave), MESOR (the value midway between the highest and lowest values of the cosine wave), and amplitude (the difference between the maximum height of the wave and the rhythm-adjusted mean [MESOR] of the wave form). We obtained values for the rhythmic parameters: MESOR, amplitude and acrophase for each day (when the distribution was adjusted to a cosine wave with a period of 24 h). The periodograms (distribution of all periods in the temporal series to determine the presence of a predominant period) were generated from each period (control, diabetic and different treatments) using the Sokolov-Bushell method (El Temps® software).

### Experimental design

**Figure 1:**
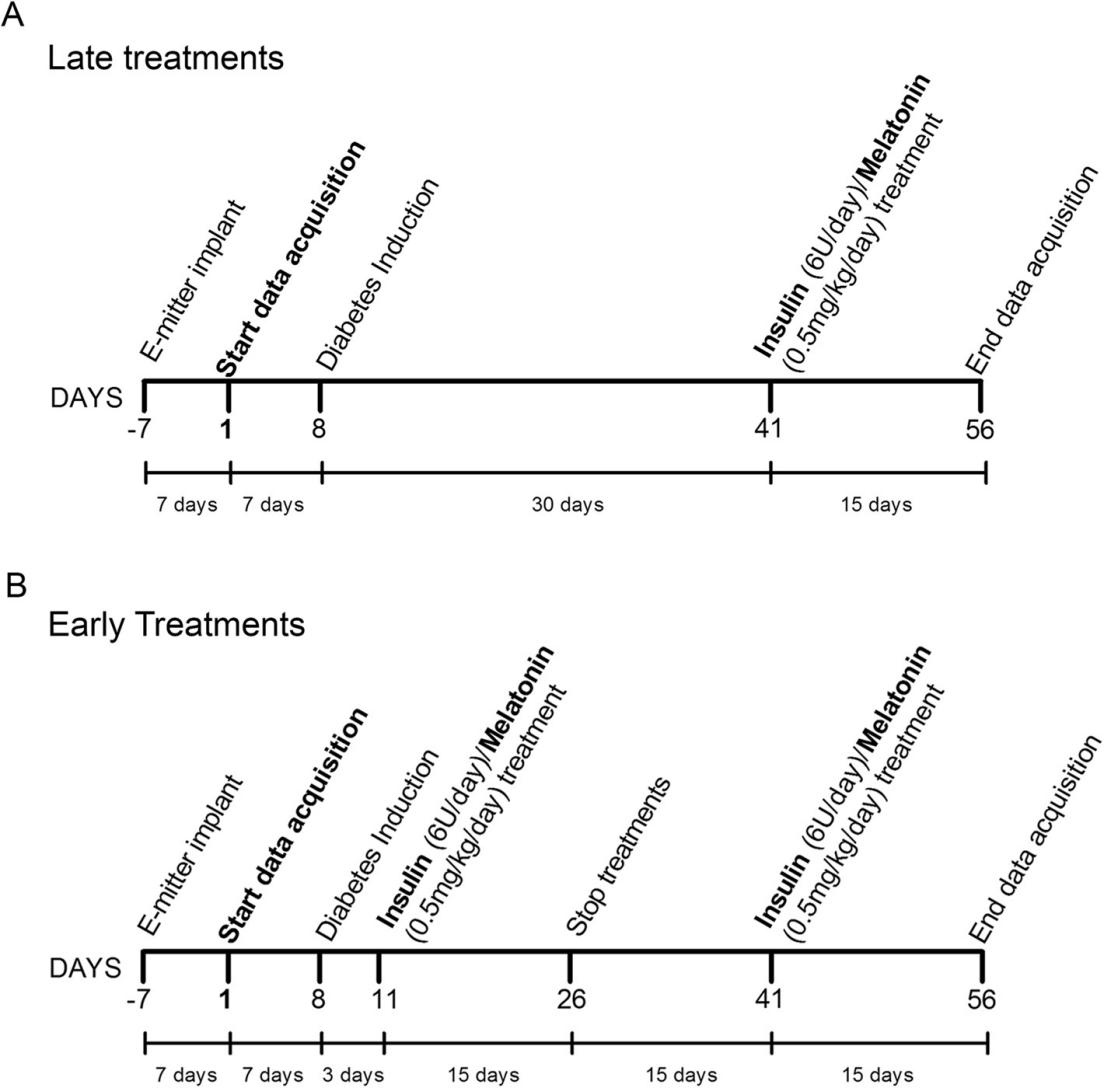
Experimental design. Young male rats were entrained to a 12:12 LD photoperiodic cycle for two weeks. Then, they were surgically implanted with E-mitter probes and their body temperature rhythm was recorded for 1 week every 30 seconds. After that, they were diabetic induced with streptozotocin and separated in two large groups: **A)** late treated and **B)** early treated with insulin (INS), melatonin (MEL) or a combination of both (INS + MEL). The first group started their treatment 33 days after STZ injection for fifteen days; the second group (B) begun the treatments three days after STZ, for fifteen days. After that, the treatments were interrupted and their rhythms recorded for other fifteen days. At day 41, treatments were resumed for fifteen more days.

After the recovering period, we started data acquisition and recorded the control period (7 days) of BT prior to STZ injection. The diabetic animals were randomly divided into two groups, the late treated group and the early treated group. The late treated group received insulin (late-INS), melatonin (late-MEL) or insulin + melatonin (late-INS + MEL) 33 days after STZ-injection (Figure 1A). The early treated group received insulin (early-INS), melatonin (early-MEL) or insulin + melatonin (early-INS + MEL) starting on the 3rd day after STZ-injection and sustained for the following 15 days. Then, the treatments were interrupted and reinstituted 15 days later (Figure 1B). The purpose of using a within-animal control was to minimize inter-individual variability in the assessment of the effects of long-term and short-term diabetes on the BT daily rhythm and the consequences of late and early treatments. Therefore, each rat acted as its own control.

### Statistical analysis

Data were classified by groups and expressed as mean ± SEM. One-way ANOVA analysis was performed to determine variation in data throughout 24 h. Statistical significance was set at p < 0.05. The temporal series were globally analyzed and rhythmic statistics performed using COSANA software applying the Cosinor method for rhythmic analyses. MESOR, amplitude and acrophase were only considered for linear analysis in days where one-way ANOVA were significant. Statistical analyses were performed using the GraphPad PRISM software (GraphPad Software, CA, USA).

## Results

For each group (n = 3) a representative animal is shown in rhythmic analyses, given that each one acted as its own control. Thermograms for all the animals are shown in Additional file 1 (late treatment) and Additional file 2 (early treatment). It should be stressed that all the effects described for the representative animal were observed in the whole group (Additional files 1 and 2).

### STZ- induced diabetes disrupts the body temperature daily rhythm

**Figure 2:**
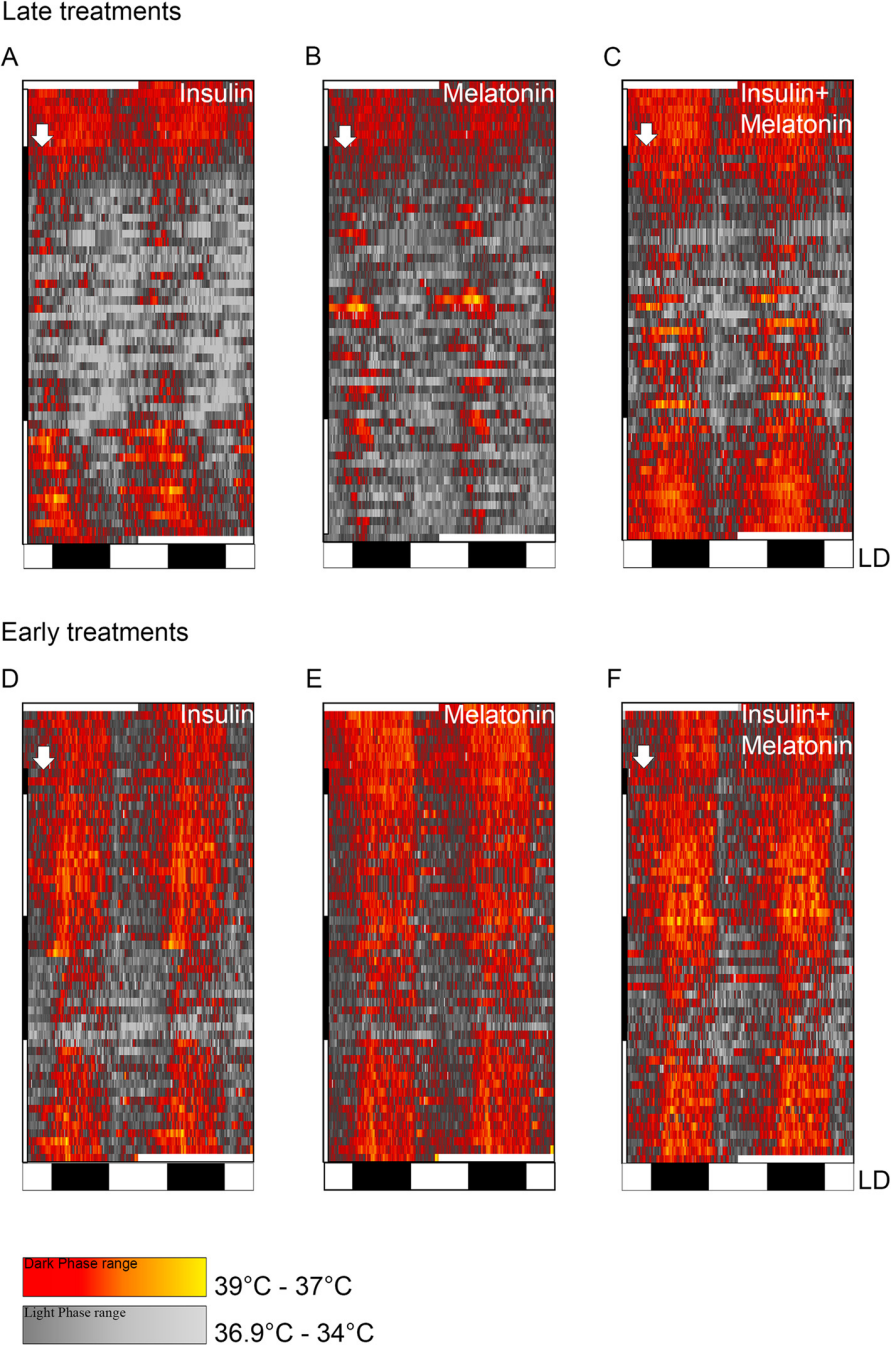
Daily rhythm of body temperature in diabetic animals. Representative double-plot thermograms of BT in late-treated animals (top row) and early-treated animals (bottom row) with insulin **(A**,** D)**, melatonin **(B**,** E)** or a combination of both hormones **(C**,** F)**. Red to yellow coloration indicates higher temperatures and black to grey coloration indicates lower temperatures throughout 55 days of record in 30 seconds bins. Black and white rectangles under the thermograms indicate the LD cycle maintained during the experiments. Late treated animals, **(A**-**C)**: control period (lateral top white bar), long-term diabetic period (lateral black bar), and treated period (bottom white bar). Early treated animals **(D**-**F)**:short-term diabetic period (lateral top black bar), early treatment (top white bar), off-treatment period (bottom black bar) and restituting treatment period (bottom white bar). The white arrow indicates the moment of the STZ injection.

**Figure 3:**
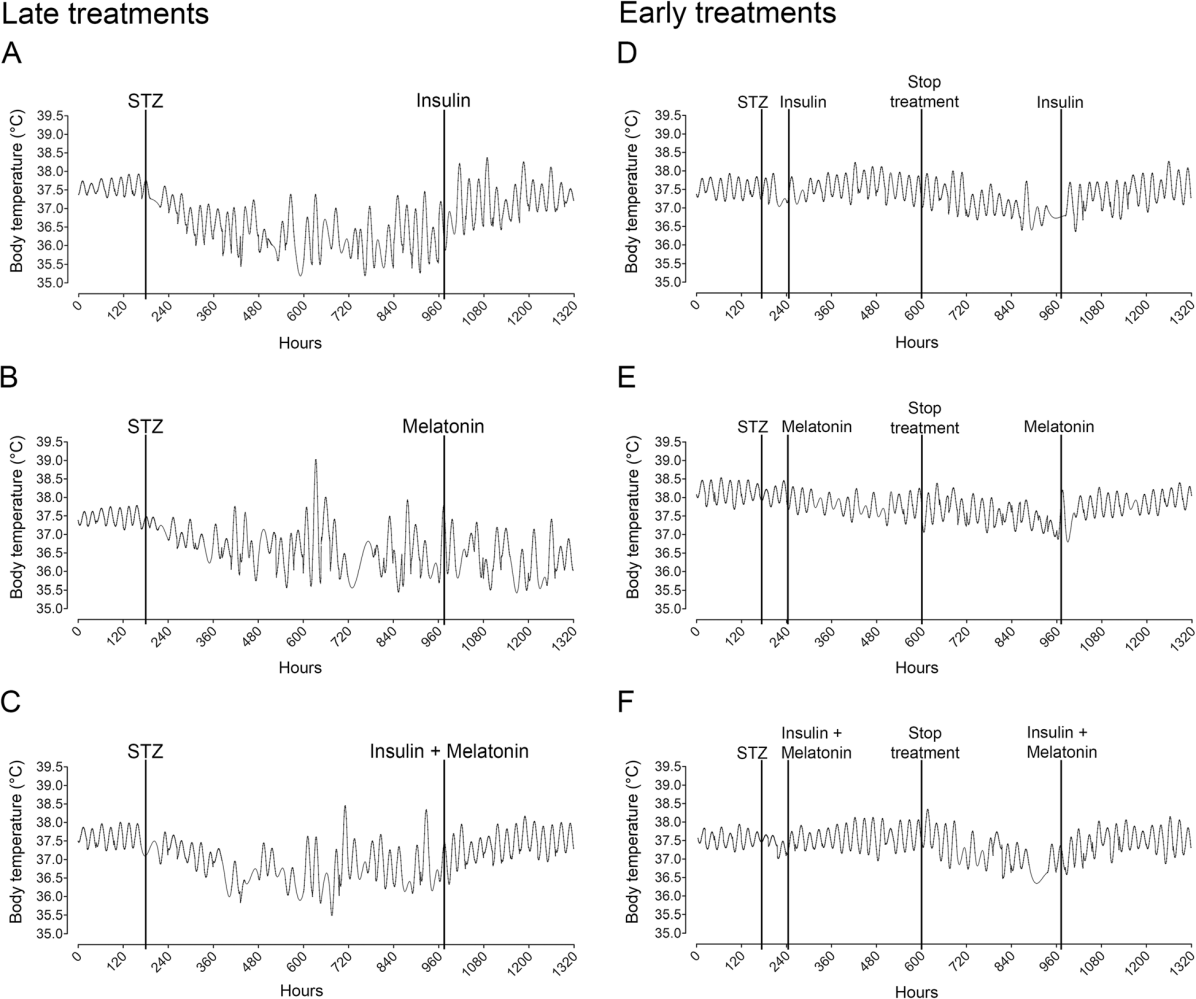
Cosine adjusted curve of late-treated and early-treated diabetic animals. Diabetic animals received late treatment (left column) or early treatment (right column) with insulin **(A**,** D)**, melatonin **(B**,** E)** or a combination of both hormones **(C**,** F)**. The cosine adjusted curve was obtained with Cosinor analyses for each day of the recording of each animal. The resulting data for each day (24 bins) was plotted throughout the recording (55 days). Only days that showed a 24 h oscillation in the Cosinor analysis were used. Lines indicate the moments of the STZ injection, the treatments and their interruption.

Under LD cycle rats exhibit well-defined 24 h rhythm of body temperature with higher temperatures at nighttime (dark phase) and lower temperatures at daytime (light phase) (Additional file 3). Since BT rhythm is considered a marker rhythm for the circadian system, we evaluated the effects of long-term diabetes (33 days after STZ injection) on the BT rhythmical structure and on its rhythmic parameters (acrophases, MESOR and amplitudes). Overall analysis (33 days’ time series) of long-term untreated diabetic rats showed alterations in their 24 h BT rhythm, characterized by phase shifts (advances and delays), and eventually days with no 24 h rhythm (Figure 2A-C). During long-term diabetes no period in the range of 20–28 h was statistically predominant; meaning that body temperature lost its circadian rhythmicity (Additional file 4B, E, and H). The day-by-day analysis of the same time series of 33 days, using the Cosinor method, showed that all rhythmic parameters were significantly altered. The daily analysis showed that the majority of the acrophases were advanced, the MESORs reduced and amplitudes increased (Table 1 and Figure 3A-C).

**Table 1:** **Average rhythmic parameters in insulin, melatonin and insulin plus melatonin late-treated diabetic animals**

	**Insulin treated group**		
	**Control**	**Diabetic**	**Insulin**
MESOR	37.59 ± 0.01487	36.48 ± 0.07885^***^	37.22 ± 0.07453^aaa^
Amplitude	0.2300 ± 0.02289	0.5556 ± 0.03913^***^	0.5521 ± 0.05900^**^
Acrophase	13.45 ± 0.9341	10.58 ± 0.4889^*^	9.762 ± 0.6649^**^
	**Melatonin supplemented group**		
	**Control**	**Diabetic**	**Melatonin**
MESOR	37.47 ± 0.01503	36.75 ± 0.07445^***^	36.48 ± 0.07706^***^
Amplitude	0.2357 ± 0.01950	0.555 ± 0.06827^*^	0.5917 ± 0.06674^*^
Acrophase	14.59 ± 0.4758	11.04 ± 0.7539	11.59 ± 0.9328
	**Insulin + melatonin treated group**		
	**Control**	**Diabetic**	**Insulin + Melatonin**
MESOR	37.62 ± 0.01857	36.96 ± 0.06499^***^	37.38 ± 0.05622^aaa^
Amplitude	0.3057 ± 0.02103	0.5291 ± 0.05421^*^	0.4533 ± 0.03055
Acrophase	14.65 ± 0.4103	13.65 ± 0.4664	13.90 ± 0.2138

ONE-Way ANOVA with *p* < 0.05 for the treatment factor followed by Bonferroni post-test. ***P < 0.001, **P < 0.01, *P < 0.05 vs Control, ^aaa^ *P* < 0.001 vs Diabetic, ***p < 0.001, *p < 0.05 vs Control, ***p < 0.001, *p < 0.05 vs Control, ^aaa^ *p* < 0.001 vs Diabetic. Mean ± SEM.

### After long-term diabetes insulin treatment restores BT rhythm and is more efficient when combined with melatonin

**Figure 4:**
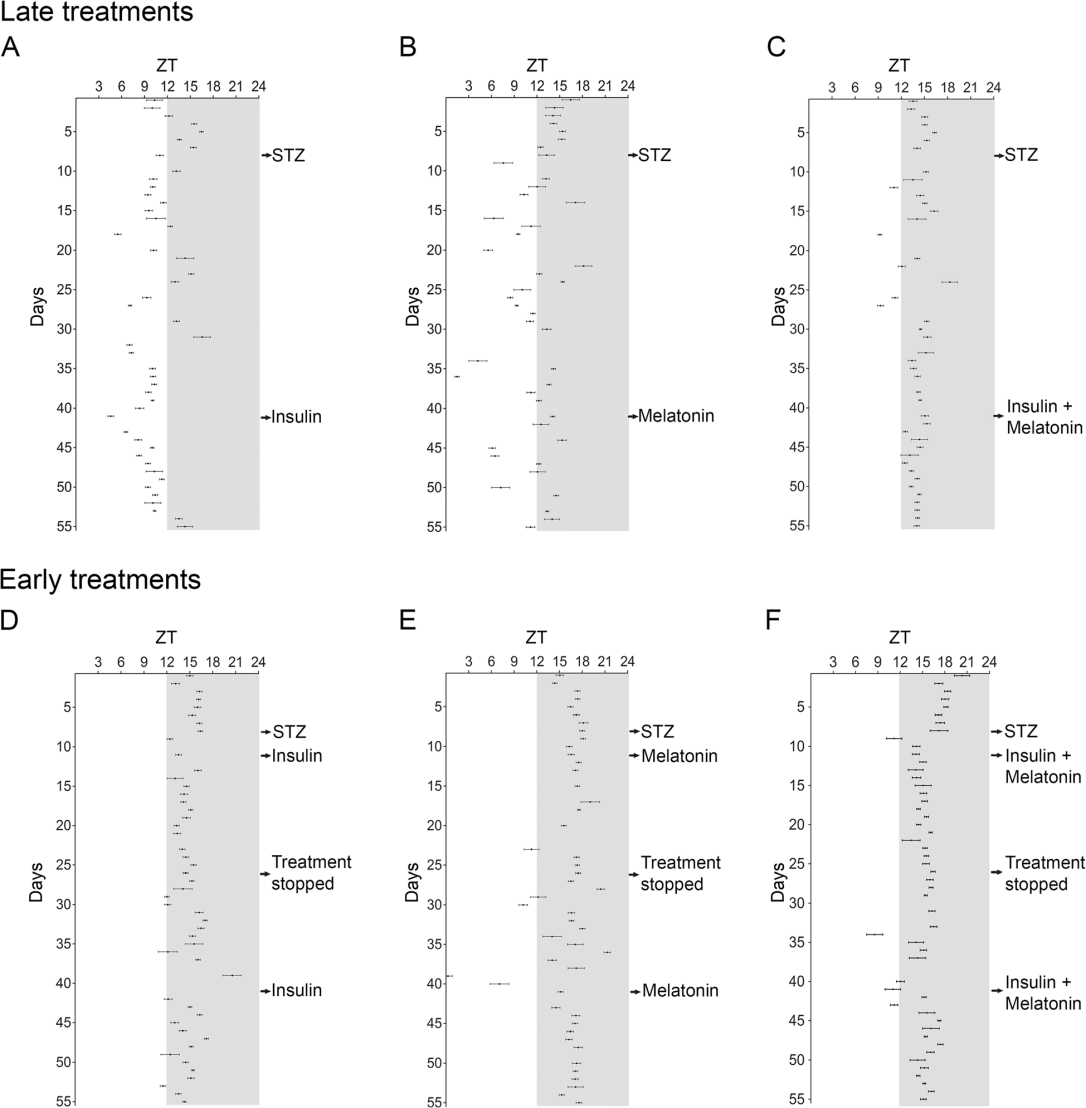
Acrophase map of late-treated and early-treated diabetic rats. Diabetic animals received late treatments (top row) or early treatments (bottom row) with insulin **(A**,** D)**, melatonin **(B**,** E)** or a combination of both hormones **(C**,** F)**. To generate the map, acrophases resulting from day-to-day analyses (averaged in 1 day bins) that showed 24 h oscillations were plotted according to the indicated ZT to generate the daily pattern vertically (mean ± SEM). Arrows indicate the days of STZ induction, treatments and their interruption.

**Figure 5:**
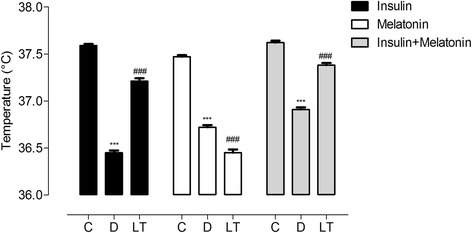
Histogram of mean BT for Late-INS, Late-MEL and Late-INS + MEL treated diabetic animals. ONE-way ANOVA with *p* < 0.0001 for treatment factor followed by Bonferroni post-test. ***p < 0.001 vs CT, ^###^ *p* < 0.001 vs D. Mean ± SEM.

We assessed the efficiency of treatments with insulin, melatonin and a combination of both hormones introduced 33 days after the establishment of the diabetic metabolic state on reversing the disruption of the BT rhythm described before. Insulin restored the daily rhythmicity (Figure 2A) and MESOR (Additional file 5A and Table 1). However it had no effect on the phase advance of the rhythm (Figure 4A) or the increased amplitude (Additional file 6A and Table 1). This resulted in a different BT rhythm contrasting with its control period (Figure 3A). During the treatment the animals returned to normoglycemia (Additional file 7) and continued to gain weight (Additional file 8). Differently from observed with insulin, the disruption of the rhythm was maintained despite melatonin supplementation (Figure 3B). Between days 25–30, in this representative animal, there were specific days with BT higher than control period, due to the alteration of the rhythm (Figure 2B). Disorganization in the acrophase map remained (phase advances, phase delays and days without 24 h rhythm) (Figure 4B), and, in average, acrophases were advanced, although they did not reach statistical significance (Table 1). MESOR remained reduced (Additional file 5B and Table 1) and amplitudes increased (Additional file 6B and Table 1). INS + MEL treatment restored 24 h BT rhythm (Figure 2C and 3C). The acrophase map was restored as in control period, since the acrophases during the treatment returned to the dark phase (Figure 4C). Both MESOR and amplitude were restored to normal values (Additional file 5C, Additional file 6C and Table 1). In conclusion, the daily pattern of BT rhythm was restored, being INS + MEL the most efficient treatment since the rhythm was synchronized even more than it was during the control period (Figure 3A-C). Late-INS and late INS + MEL treatments reversed the reduction in mean BT; however neither of them reached statistical significance (Figure 5). All late treatments restored the predominant period of 24 h, lost with the progression of the disease (Additional file 4). As expected, treatments where insulin was administered resulted in weight gain and restoration of normoglycemia; melatonin supplementation led to a slight weight gain but had no effect on hyperglycemia (Additional files 7 and 8).

### Early treatments prevent the disruption of the BT rhythm caused by diabetes; melatonin supplementation maintains BT rhythm synchronized. Insulin and melatonin combined is most efficient. Beneficial effects are suppressed when the treatment stops

**Figure 6:**
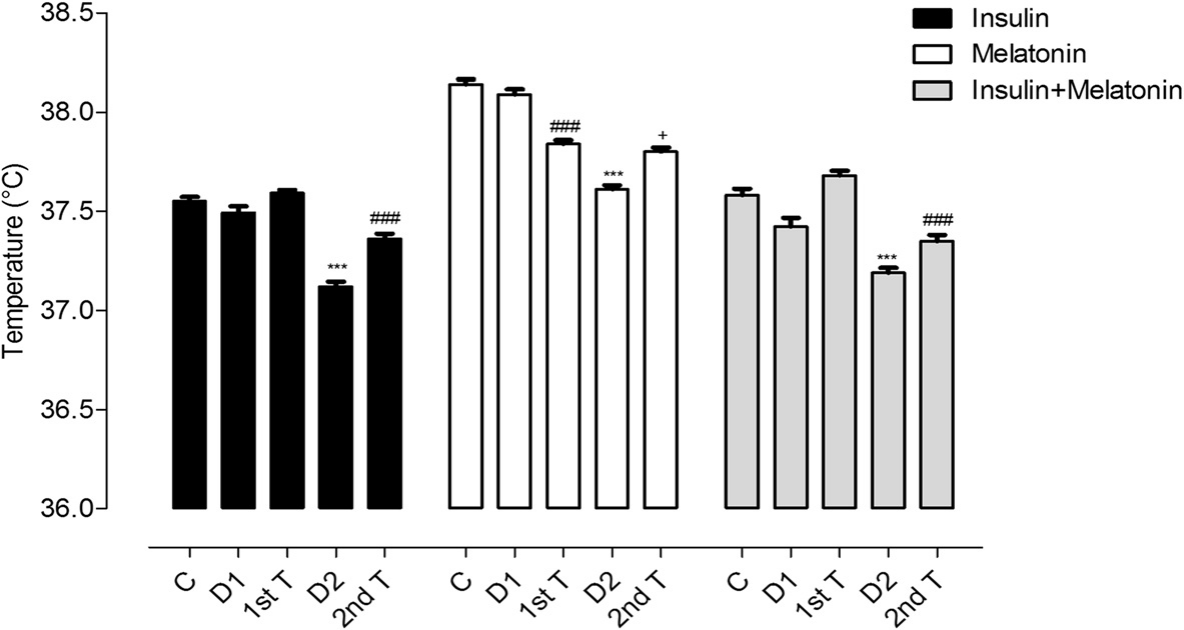
Histogram of mean BT for Early-INS, Early-MEL and Early-INS + MEL treated diabetic animals. ONE-Way ANOVA with *p* < 0,0001 for treatment factor followed by Bonferroni post-test. ***p < 0.001 vs all groups, ^###^ *p* < 0,001 vs all groups..***p < 0.001 vs CT, D1 and D2. **p < 0.01 vs all groups, ^###^ *p* < 0,001 vs CT, DIM1 and D2, ^aaa^ *p* < 0,001 vs D. Mean ± SEM.

In order to study whether the treatments are able to prevent the effects observed in the temperature rhythm in long-term diabetes, three groups of animals were treated 3 days after STZ injection for 15 days. Early-INS and early-INS + MEL groups showed normal BT rhythmicity, and early-MEL showed some alterations in it (Figure 2D-F and 3D-F). To assess which components of the rhythm are restored with the early treatments and which ones remain altered we analyzed the presence of a 24 h period, MESOR, acrophases and amplitudes. All early treatments prevented the loss of the 24 h period (Additional file 9). During the first 15 days of treatment with insulin and insulin plus melatonin, the animals presented a 24 h rhythm with the acrophases allocated in the dark phase. Early-MEL rats showed few days without 24 h rhythm and some days with phase advances, particularly towards the end of the treatment (Figure 4E). MESOR was maintained in early-INS and early-INS + MEL but not in early-MEL rats, where a progressive decrease of the daily mean temperature was observed (Additional file 5D-F and Table 2). The amplitude of the rhythm increased in early-INS + MEL and early-MEL rats during the 15 days of treatment but did not in early-INS rats where the amplitude remained as observed during the control period (Additional file 6D-F). INS and INS + MEL early treatments prevented the reduction in BT seen in long-term diabetic rats, maintaining normal BT; differently, early-MEL rats showed a significant reduction in BT (Figure 6). In conclusion, BT daily rhythm was maintained with all early treatments, being INS more effective than MEL and the combination of both hormones the most effective treatment to preserve the rhythm as a whole (Figure 3D-F). As expected, early-INS and early-INS + MEL rats were normoglycemic and early-MEL rats remained hyperglycemic during treatments (Additional file 7D-F). The increase in body weight was more significant in animals that received insulin when compared with those that received only melatonin (Additional file 8D-F).

**Table 2:** **Average rhythmic parameters in insulin, melatonin and insulin plus melatonin early-treated diabetic animals**

	**Insulin treated group**			
	**Control**	**1st Insulin**	**Diabetic**	**2nd Insulin**
MESOR	37.55 ± 0.01850	37.59 ± 0.02640	37.18 ± 0.06034^***bbb^	37.41 ± 0.05469^aab^
Amplitude	0.2814 ± 0.01969	0.3546 ± 0.0248	0.3915 ± 0.042	0.4329 ± 0.0908^*^
Acrophase	15.46 ± 0.4256	14.31 ± 0.2471	15.18 ± 0.6522	14.27 ± 0.4303
	**Melatonin supplemented group**			
	**Control**	**1st Melatonin**	**Diabetic**	**2nd Melatonin**
MESOR	38.15 ± 0.01875	37.84 ± 0.02414^***^	37.57 ± 0.05185^***bbb^	37.84 ± 0.03808^***aaa^
Amplitude	0.3157 ± 0.02467	0.3460 ± 0.02156	0.3473 ± 0.02697	0.3477 ± 0.02822
Acrophase	16.57 ± 0.5227	16.66 ± 0.6569	14.60 ± 1.400	16.57 ± 0.2733
	**Insulin + Melatonin treated group**			
	**Control**	**1st Insulin + Melatonin**	**Diabetic**	**2nd Insulin + Melatonin**
MESOR	37.55 ± 0.01672	37.58 ± 0.01696^*^	37.29 ± 0.08757^bb^	37.44 ± 0.04681
Amplitude	0.2543 ± 0.02553	0.3740 ± 0.02801	0.4218 ± 0.03670^*^	0.4127 ± 0.03099^*^
Acrophase	18.08 ± 0.4162	14.89 ± 0.1850^***^	14.63 ± 0.7259^***^	15.06 ± 0.4708^**^

ONE-Way ANOVA with *p* < 0.05 for the treatment factor followed by Bonferroni post-test. ***p < 0.001, **p < 0.01, *p < 0.05 vs Control, ^aaa^ *p* < 0.001,^aa^ *p* < 0.01 vs Diabetic, ^bbb^ *p* < 0.001, ^bb^ *p* < 0.01,^b^ *p* < 0.05 vs 1st Insulin, ***p < 0.001 vs Control, ^aaa^ *p* < 0.001 vs Diabetic, ^bbb^ *p* < 0.001 vs 1st Melatonin, ***p < 0.001, **p < 0.01, *p < 0.05 vs Control, ^bb^ *p* < 0.01 vs 1st Insulin + Melatonin. Mean ± SEMn.

To study whether the preventive effects showed above are dependent on the continuity of the treatments, after 15 days they were interrupted for two weeks and the rhythmic effects were evaluated.

All animals presented a 24 h period of the temperature rhythm; however, they presented a decreased potency (Qp) on their periodograms (Qp = 150–200 during early treatments vs 80–100 when treatments were interrupted) (Additional file 9 second column for treated period and third for interrupted period). The interruption of the treatments led to a severe alteration in the rhythm (Figure 3D-F). All three groups showed a delay in the onset of the alterations following the interruption, and it was more evident in early-INS + MEL group, probably due to a protective effect of the early treatments (Figure 2D-F). Early-INS and MEL rats showed alterations in their acrophase maps being more evident in early-MEL; however this alteration did not reach statistical difference (Table 2). Despite the altered acrophases (phase delays and advances), they were mostly in the dark period (Figure 4D-F). MESOR was reduced progressively (Additional file 5D-F and Table 2) and amplitude showed no significant alteration when compared to the previous period (Additional file 6D-F and Table 2). During the interruption BT was significantly reduced in all three groups, although in a minor magnitude when compared with long-term diabetic animals, indicating that all early treatments in some way prevented a bigger reduction of body temperature (Figure 6). Also, during the interruption, hyperglycemia was rapidly established and the animals lost weight (Additional files 7 and 8). In conclusion, the interruption of the treatments led to the loss of their effects on the rhythm; however during that period the rhythm was not as disrupted as seen in long-term diabetic animals. The most efficient early treatment was INS + MEL; it preserved the structure of the rhythm for 5 days before the effects of diabetes were established (Figure 3D-F).

Finally, we studied if the interruption leaves consequences in the daily BT rhythm when the treatments are reinstituted. After reinstitution, daily rhythmicity was rapidly restored in all groups (Figure 2D-F), and the acrophases returned to the dark phase, being more synchronized in those groups where melatonin was administered (Figure 4D-F and Table 2). The potency of the 24 h period was restored as in control and early treated period (Additional file 9D, H, and L). After reestablishment of the treatments, only those groups where insulin was administered showed a restored MESOR. In early-MEL rats MESOR attained the pattern and values of the early supplementation (Additional file 5D-F and Table 2). The amplitude remained increased in early-INS and early-INS + MEL rats but not in early-MEL rats, where it remained unaltered (Additional file 6D-F and Table 2). In conclusion, the pattern of the daily rhythm of BT was restored, with higher temperatures at nighttime and lower temperature at daytime; however, early-MEL rats showed improved synchronization of the rhythm, when compared with early-INS rats. Finally, early-INS + MEL rats had the most synchronized rhythm among the three, but the restitution was not as efficient as the early treatment (Figure 3D-F). As expected, body weight and glycemia were normalized with restitution in early-INS and early-INS + MEL groups but not early-MEL (Additional files 7 and 8).

## Discussion

STZ-induced diabetes disrupts body temperature daily rhythm, and alters all its rhythmic parameters (MESOR, amplitude and acrophase). Given its structure, BT is known and widely considered one of the “marker rhythms” of the biological clock. Its complex chronobiological, neural, endocrine, metabolic and molecular regulation involves several metabolic elements, such as thyroid hormone, brown adipose tissue (BAT), leptin and hypothalamic neural structures, in particular, the suprachiasmatic nuclei.

It is well documented that STZ-induced diabetes causes a significant decrease in mean BT [29,32,33], and that exogenous insulin treatment is able to restore it [30]. In this study we describe for the first time, to our knowledge, the daily profile of BT in a insulin-deficient diabetic animal, and we demonstrate that diabetes not only causes a significant reduction in BT, but also the disruption of its daily rhythm (no rhythms with period between 20 and 28 hours), disturbance that intensifies with time, showing alterations in all rhythmic parameters, such as decreased MESOR, increased amplitude and shifted acrophases.

In the present experimental design, the results of the rhythmic analysis came from the same animal as its own control, given that each individual has a particular daily profile and range of BT, which would be lost if a pool of animals were used instead.

Diabetes is a metabolic pathology directly related to energy metabolism and, consequently, temperature regulation. The thyroid gland is an important gland for thermoregulation, and it is reported the occurrence of hypothyroidism associated with most common experimental type 1 diabetes models (STZ and alloxan), [42-47]. More recent data shows that STZ- induced diabetes leads to a true hypothyroidism (decreased free T3 and T4 levels, along with clinical signals for hypothyroidism, and increased Q-T interval). Exogenous insulin treatment prevents all signs of hypothyroidism and restores normal free T3 and T4 levels; T3 treatment alone normalizes BT, heart rate and Q-T interval, but has no effect on glycemia [48,49], therefore the reduction of the mean body temperature observed in our work would probably be result of a hypothyroidism secondary to STZ-induced diabetes.

Other studies demonstrate the importance of BAT in regulation of BT. BAT thermogenesis contributes to the increase of core BT during the dark phase, indicating that circadian changes of BAT thermogenesis does indeed play significant role in the overall maintenance of the circadian rhythm of core BT [50].

Another important consequence of insulin dependent diabetes in rodents is leptin deficiency [51]. It has been demonstrated that diabetic animals also have decreased leptin, and that insulin treatment is able to restore it. Since leptin is a major regulator of thermoregulation and energy metabolism through the balance of body weight and adipose tissue [52], an important feature of the loss of thermoregulation in STZ-induced diabetic animals could be the reduction of leptin levels. It would be important to assess the leptin levels in both groups, in short-term and long-term to determine leptin’s part in regulating the body temperature rhythm.

Previous work shows that in experimental diabetes, a significant decrease of BT occurs only eight weeks after STZ injection and that two weeks are not enough to observe this change [48]. These data differs from other in literature [29,33], and from the present work, where a significant decrease in core BT is evident two weeks after diabetes induction. This discrepancy is probably due to methodological differences, given that Zhang et al. measured rectal BT only twice (two and eight weeks after STZ), instead of monitoring it continuously, as was done in the present study. Moreover, as it was shown in the temporal analysis of the present data, even though the BT rhythm is disrupted and, in average, temperature is decreased, at some points temperature still reaches normal values and therefore few samples of rectal BT would not necessarily detect the mean reduction in BT caused by diabetes, already seen at the onset of the disease.

Melatonin is the hormone that signalizes exterior daytime and nighttime to the body, and is also important to maintain internal synchronization [20]. Previous work form our lab demonstrated that during T1DM rats have significant reduction of melatonin synthesis both in the pineal gland [38] and in the retina [39]. In the present study, short-term diabetes was sufficient to disturb the BT rhythmicity even though the 24 h period remained, indicating that three-day diabetes is enough to produce circadian alterations. In accordance with these evidence, and because melatonin is a major regulator of BT rhythm in humans [53]; we supplemented diabetic animals with melatonin, in physiological doses. Melatonin alone did not restore the rhythmic parameters, or reverse the reduction of BT in long term diabetic animals. When administered early, melatonin partially maintained BT rhythmicity although it was not as efficient as insulin, since MESOR was significantly reduced, and animals remained hyperglycemic and stopped gaining weight. However, melatonin had an effect on the regulation of BT since a further decrease was observed when the supplementation was interrupted. Some characteristics of the long-term diabetic animals appeared. After the reinstitution of the supplementation all animals returned to the same state as they were during the first supplementation. Based on these results, early melatonin ameliorates the effects of diabetes on BT rhythm. Melatonin seems to have a stronger effect on rhythmicity, while insulin seems to act both on the metabolic and chronobiological effects of diabetes. Still, we demonstrated here that melatonin early treatment is important to prevent the disruption in the BT rhythm caused by diabetes. In fact, it was demonstrated that in STZ-diabetic animals pineal melatonin production is reduced by more than 50% [38] and that melatonin is important for the integrity and function of the BAT [25] as well as for the daily distribution of thermogenic processes [54]. The absence of melatonin reduces nighttime energy expenditure and temperature. Taking together these data, it should be postulated that the reduction in melatonin associated to the STZ-induced diabetes is responsible at least partially for the rhythmic disturbances observed in body temperature. This result in some way diverges from data in literature showing a hypothermic effect of melatonin treatment [55], where they used pharmacological doses (30-120 mg/kg i.p.) in healthy animals whereas this study studied a near physiological dose (0.5 mg/kg in drinking water) in diabetic animals.

A combination of melatonin plus insulin restored normal BT and its rhythm, including daily allocation of the acrophases at night and normal MESOR and amplitudes. These results show a complementary action of insulin and melatonin to restore BT, body weight, glycemia and the daily rhythm in long-term diabetic animals. The early treatment of diabetic animals with insulin plus melatonin increased BT above control values and maintained its rhythm. This could be explained by a combined action of melatonin and insulin on BT, since they both act increasing it. When the treatments were interrupted, some of the effects observed during the treatments were lost. Reinstituting treatments restored MESOR, but not amplitude nor acrophases. These results suggest that 15 days of diabetes, even after receiving treatment, lead to persistent alterations of BT rhythm and all rhythmic parameters, along with body weight loss and hyperglycemia, and the reinstitution of the treatments does not result as effective as treating the animals after an early onset. They also suggest a possible interaction between insulin and melatonin in diabetic animals that leads to a more efficient treatment of the disruption in the daily rhythm of BT. Since body temperature is a marker rhythm of the state of internal synchronization of the body, the alteration of its structure probably leads to a strong alteration in the synchronization of the entire body. Another factor to be considered is a progressive and severe, insulin resistance [56].

We still need to study the mechanisms underlying this phenomenon; however, we suppose that melatonin potentializes insulin action in BT rhythmicity acting centrally [57] or peripherally [58]. In this way, it could be of great importance to include melatonin as a complement in insulin treatment of diabetes, given that disruption of the BT rhythm, and probably other rhythms in the organism are not completely reverted by insulin alone.

Despite the alterations in the rhythmic parameters seen after 3 and 15 days of diabetes, neither of them was sufficient to cause the loss of the 24 h period, differently from what was observed with 33 days, where the all periods - from 20 to 28 h - were lost. All treatments were able to prevent the loss of the 24 h predominant period; however a reduction in the potency of the predominance of the 24 h period was evident in every case. It seems that the 24 h period is a characteristic of the circadian system that persists even when other rhythmic parameters are disrupted and, different from MESOR, amplitude and acrophase, it was completely restored by all late treatments.

This study shows for the first time, to our knowledge, the alterations in the daily rhythm of BT, one of the most important rhythms, caused by STZ-induced diabetes. It is important to know that diabetes not only causes metabolic damages, but it also damages the circadian system; this information is relevant for a wider approach when designing new therapies for treating this disease, considering a new system that results significantly altered and is not fully restored by therapies in use. More studies are needed to understand the mechanisms behind these observations, such as the role of BAT and thyroid gland on the BT rhythm in this model, and integrate the chronobiological, metabolic, neural systems.

## Conclusions

In conclusion, we describe a new consequence of insulin dependent diabetes: the disruption of the BT rhythm; when the disease is treated, insulin can restore MESOR and amplitude, but with peaks of BT in the light phase; melatonin is not able to either restore the rhythm or reverse the decreased BT; joint treatment of insulin and melatonin restores average BT and its normal daily rhythm. Early treatments can prevent the disruption of the rhythm. Melatonin as an early treatment, acts partially increasing BT. Insulin and melatonin appear to act in a complementary way restoring the rhythm. All beneficial effects observed are dependent on the continuous maintenance of the treatments and; reinstituting treatments do not result as effective as the early ones. Melatonin seems to improve insulin action restoring the endogenous rhythm of body temperature after long-term diabetes. Therefore, melatonin could be a new complement of insulin treatment improving its action on the energy metabolism.

## Additional files

Additional file 1:
**Representative 48 h individual body temperature profiles in** **Late-INS** **(A),** **Late-MEL** **(B), and** **Late-INS** **+ MEL (C) animals.**
Additional file 2:
**Representative 48 h individual body temperature profiles in** **Early-INS** **(A),** **Early-MEL** **(B) and** **Early-INS** **+ MEL (C) animals.**
Additional file 3:
**Representative 48 h individual body temperature profiles in Control animals.** Citrate buffer has no effect on body temperature profiles. Additional file 4:
**Representative periodograms for** **Late-INS** **(A-C),** **Late-MEL** **(D-F) and** **Late-INS** **+ MEL (G-I) animals indicating the predominant period of the control (A, D, G), diabetic (B, E, H) and treated phases (C, F, I).** The threshold line indicates when a determined period was statistically predominant in the temporal series. Additional file 5:
**Temperature MESOR resulting from** **day-to-day** **analyses of BT averaged in 1 h bins in late treatments (A-C) and early treatments (D-F) with insulin (A, D), melatonin (B, E) and insulin plus melatonin (C, F).** Only days that showed 24 h oscillations were considered. Additional file 6:
**Amplitude of the BT rhythm resulting from** **day-to-day** **analyses averaged in 1 h bins in late treatments (A-C) and early treatments (D-F) with insulin (A, D), melatonin (B, E) and insulin plus melatonin (C, F).** Only days that showed 24 h oscillations were considered. Additional file 7:
**Variations in glycemia averaged per week in diabetic animals treated with insulin (A, D), melatonin (B, E) or insulin plus melatonin (C, F).** Upper row shows late treatments and bottom row, early treatments. Mean ± SEM. Additional file 8:
**Body weight of diabetic animals averaged per week treated with insulin (A, D), melatonin (B, E) and insulin plus melatonin (C, F).** Upper row shows late treatments and bottom row, early treatments. Mean ± SEM. Additional file 9:
**Representative periodograms for** **Early-INS** **(A-D),** **Early-MEL** **(E-H) and** **Early-INS** **+ MEL (I-L) animals indicating the predominant period of the control (A, E, I), early treatment (B, F, J),** **off-treatment** **(C, G, K) and restituting treatment phases (D, H, L).** The threshold line indicates when a determined period was statistically predominant in the series.

## Abbreviations

SCN: Suprachiasmatic nuclei
T1DM: Type 1 diabetes mellitus
STZ: Streptozotocin
BT: Body temperature
ZT: Zeitgeber time
MESOR: Midline estimating statistic of rhythm
INS: Insulin
MEL: Melatonin
INS + MEL: Insulin and melatonin
LD: Light and dark cycle
BAT: Brown adipose tissue

## References

[1] Karatsoreos IN, Bhagat S, Bloss EB, Morrison JH, McEwen BS (2011). Disruption of circadian clocks has ramifications for metabolism, brain, and behavior. Proc Natl Acad Sci U S A.

[2] Minors DS, Folkard S, Waterhouse JM (1996). The shape of the endogenous circadian rhythm of rectal temperature in humans. Chronobiol Int.

[3] Hanneman SK (2001). Measuring circadian temperature rhythm. Biol Res Nurs.

[4] Kelly G (2006). Body temperature variability (Part 1): a review of the history of body temperature and its variability due to site selection, biological rhythms, fitness, and aging. Altern Med Rev.

[5] Scheer FA, Pirovano C, Van Someren EJ, Buijs RM (2005). Environmental light and suprachiasmatic nucleus interact in the regulation of body temperature. Neuroscience.

[6] la Fleur SE, Kalsbeek A, Wortel J, Fekkes ML, Buijs RM (2001). A daily rhythm in glucose tolerance: a role for the suprachiasmatic nucleus. Diabetes.

[7] la Fleur SE, Kalsbeek A, Wortel J, van der Vliet J, Buijs RM (2001). Role for the pineal and melatonin in glucose homeostasis: pinealectomy increases night-time glucose concentrations. J Neuroendocrinol.

[8] Reiter RJ, Tan DX, Korkmaz A (2009). The circadian melatonin rhythm and its modulation: possible impact on hypertension. J Hypertens Suppl.

[9] Peschke E, Frese T, Chankiewitz E, Peschke D, Preiss U, Schneyer U (2006). Diabetic Goto Kakizaki rats as well as type 2 diabetic patients show a decreased diurnal serum melatonin level and an increased pancreatic melatonin-receptor status. J Pineal Res.

[10] Peschke E, Stumpf I, Bazwinsky I, Litvak L, Dralle H, Muhlbauer E (2007). Melatonin and type 2 diabetes - a possible link?. J Pineal Res.

[11] Goncharova ND, Vengerin AA, Khavinson V, Lapin BA (2005). Pineal peptides restore the age-related disturbances in hormonal functions of the pineal gland and the pancreas. Exp Gerontol.

[12] Scheer FA, Hilton MF, Mantzoros CS, Shea SA (2009). Adverse metabolic and cardiovascular consequences of circadian misalignment. Proc Natl Acad Sci U S A.

[13] Boden G, Chen X, Polansky M (1999). Disruption of circadian insulin secretion is associated with reduced glucose uptake in first-degree relatives of patients with type 2 diabetes. Diabetes.

[14] Scott EM, Carter AM, Grant PJ (2008). Association between polymorphisms in the Clock gene, obesity and the metabolic syndrome in man. Int J Obes (Lond).

[15] Woon PY, Kaisaki PJ, Braganca J, Bihoreau MT, Levy JC, Farrall M (2007). Aryl hydrocarbon receptor nuclear translocator-like (BMAL1) is associated with susceptibility to hypertension and type 2 diabetes. Proc Natl Acad Sci U S A.

[16] Marcheva B, Ramsey KM, Buhr ED, Kobayashi Y, Su H, Ko CH (2010). Disruption of the clock components CLOCK and BMAL1 leads to hypoinsulinaemia and diabetes. Nature.

[17] Turek FW, Joshu C, Kohsaka A, Lin E, Ivanova G, McDearmon E (2005). Obesity and metabolic syndrome in circadian Clock mutant mice. Science.

[18] Yoon JA, Han DH, Noh JY, Kim MH, Son GH, Kim K (2012). Meal time shift disturbs circadian rhythmicity along with metabolic and behavioral alterations in mice. PLoS One.

[19] Hasler BP, Buysse DJ, Kupfer DJ, Germain A (2010). Phase relationships between core body temperature, melatonin, and sleep are associated with depression severity: further evidence for circadian misalignment in non-seasonal depression. Psychiatry Res.

[20] Gubin DG, Gubin GD, Waterhouse J, Weinert D (2006). The circadian body temperature rhythm in the elderly: effect of single daily melatonin dosing. Chronobiol Int.

[21] Wolden-Hanson T, Mitton DR, McCants RL, Yellon SM, Wilkinson CW, Matsumoto AM (2000). Daily melatonin administration to middle-aged male rats suppresses body weight, intraabdominal adiposity, and plasma leptin and insulin independent of food intake and total body fat. Endocrinology.

[22] Alonso-Vale MI, Anhe GF, Borges-Silva C, Andreotti S, Peres SB, Cipolla-Neto J (2004). Pinealectomy alters adipose tissue adaptability to fasting in rats. Metab Clin Exp.

[23] Alonso-Vale MI, Borges-Silva CN, Anhe GF, Andreotti S, Machado MA, Cipolla-Neto J (2004). Light/dark cycle-dependent metabolic changes in adipose tissue of pinealectomized rats. Hormone and metabolic research = Hormon- und Stoffwechselforschung = Hormones et metabolisme.

[24] Muhlbauer E, Gross E, Labucay K, Wolgast S, Peschke E (2009). Loss of melatonin signalling and its impact on circadian rhythms in mouse organs regulating blood glucose. Eur J Pharmacol.

[25] Cipolla-Neto J, Amaral FG, Afeche SC, Tan DX, Reiter RJ (2014). Melatonin, energy metabolism, and obesity: a review. J Pineal Res.

[26] Krauchi K, Cajochen C, Wirz-Justice A (1997). A relationship between heat loss and sleepiness: effects of postural change and melatonin administration. J Appl Physiol (1985).

[27] Whiting DR, Guariguata L, Weil C, Shaw J (2011). IDF diabetes atlas: global estimates of the prevalence of diabetes for 2011 and 2030. Diabetes Res Clin Pract.

[28] King AJ (2012). The use of animal models in diabetes research. Br J Pharmacol.

[29] Howarth FC, Jacobson M, Naseer O, Adeghate E (2005). Short-term effects of streptozotocin-induced diabetes on the electrocardiogram, physical activity and body temperature in rats. Exp Physiol.

[30] Howarth FC, Jacobson M, Shafiullah M, Ljubisavljevic M, Adeghate E (2011). Heart rate, body temperature and physical activity are variously affected during insulin treatment in alloxan-induced type 1 diabetic rat. Physiological research / Academia Scientiarum Bohemoslovaca.

[31] Kilgour RD, Williams PA (1998). Diabetes affects blood pressure and heart rate responses during acute hypothermia. Acta Physiol Scand.

[32] Howarth FC, Jacobson M, Shafiullah M, Adeghate E (2005). Long-term effects of streptozotocin-induced diabetes on the electrocardiogram, physical activity and body temperature in rats. Exp Physiol.

[33] Howarth FC, Jacobson M, Shafiullah M, Adeghate E (2006). Effects of insulin treatment on heart rhythm, body temperature and physical activity in streptozotocin-induced diabetic rat. Clin Exp Pharmacol P.

[34] Seydoux J, Chinet A, Schneider-Picard G, Bas S, Imesch E, Assimacopoulos-Jeannet F (1983). Brown adipose tissue metabolism in streptozotocin-diabetic rats. Endocrinology.

[35] Kilgour RD, Williams PA (1996). Effects of diabetes and food deprivation on shivering activity during progressive hypothermia in the rat. Comp Biochem Physiol A Physiol.

[36] Smith OL, Davidson SB (1982). Shivering thermogenesis and glucose uptake by muscles of normal or diabetic rats. Am J Physiol.

[37] Peschke E, Bahr I, Muhlbauer E (2013). Melatonin and pancreatic islets: interrelationships between melatonin, insulin and glucagon. Int J Mol Sci.

[38] Amaral FG, Turati AO, Barone M, Scialfa JH, do Carmo Buonfiglio D, Peres R (2014). Melatonin synthesis impairment as a new deleterious outcome of diabetes-derived hyperglycemia. J Pineal Res.

[39] do Carmo Buonfiglio D, Peliciari-Garcia RA, do Amaral FG, Peres R, Nogueira TCA, Afeche SC (2011). Early-stage retinal melatonin synthesis impairment in streptozotocin-induced diabetic wistar rats. Invest Ophthalmol Vis Sci.

[40] Nishida S (2005). Metabolic effects of melatonin on oxidative stress and diabetes mellitus. Endocrine.

[41] Harkin A, O'Donnell JM, Kelly JP (2002). A study of VitalView for behavioural and physiological monitoring in laboratory rats. Physiol Behav.

[42] Sochor M, Baquer NZ, Ball MR, McLean P (1987). Regulation of enzymes of glucose metabolism and lipogenesis in diabetic rat liver by thyroid hormones. Biochem Int.

[43] Sundaresan PR, Sharma VK, Gingold SI, Banerjee SP (1984). Decreased beta-adrenergic receptors in rat heart in streptozotocin-induced diabetes: role of thyroid hormones. Endocrinology.

[44] Rodgers RL, Davidoff AJ, Mariani MJ (1991). Cardiac function of the diabetic renovascular hypertensive rat: effects of insulin and thyroid hormone treatment. Can J Physiol Pharmacol.

[45] Rondeel JM, de Greef WJ, Heide R, Visser TJ (1992). Hypothalamo-hypophysial-thyroid axis in streptozotocin-induced diabetes. Endocrinology.

[46] der Elst JP S-v, van der Heide D (1992). Effects of streptozocin-induced diabetes and food restriction on quantities and source of T4 and T3 in rat tissues. Diabetes.

[47] Katovich MJ, Marks KS, Sninsky CA (1993). Effect of insulin on the altered thyroid function and adrenergic responsiveness in the diabetic rat. Can J Physiol Pharmacol.

[48] Zhang L, Parratt JR, Beastall GH, Pyne NJ, Furman BL (2002). Streptozotocin diabetes protects against arrhythmias in rat isolated hearts: role of hypothyroidism. Eur J Pharmacol.

[49] Matsen ME, Thaler JP, Wisse BE, Guyenet SJ, Meek TH, Ogimoto K (2013). In uncontrolled diabetes, thyroid hormone and sympathetic activators induce thermogenesis without increasing glucose uptake in brown adipose tissue. Am J Physiol Endocrinol Metab.

[50] Yang YL, Shen ZL, Tang Y, Wang N, Sun B (2011). [Simultaneous telemetric analyzing of the temporal relationship for the changes of the circadian rhythms of brown adipose tissue thermogenesis and core temperature in the rat]. Zhongguo ying yong sheng li xue za zhi = Zhongguo yingyong shenglixue zazhi =Chinese journal of applied physiology.

[51] Havel PJ, Uriu-Hare JY, Liu T, Stanhope KL, Stern JS, Keen CL (1998). Marked and rapid decreases of circulating leptin in streptozotocin diabetic rats: reversal by insulin. Am J Physiol.

[52] Rezai-Zadeh K, Munzberg H (2013). Integration of sensory information via central thermoregulatory leptin targets. Physiol Behav.

[53] Cagnacci A, Elliott JA, Yen SS (1992). Melatonin: a major regulator of the circadian rhythm of core temperature in humans. J Clin Endocrinol Metab.

[54] Teodoro BG, Baraldi FG, Sampaio IH, Bomfim LH, Queiroz AL, Passos MA (2014). Melatonin prevents mitochondrial dysfunction and insulin resistance in rat skeletal muscle. J Pineal Res.

[55] Lin MT, Chuang JI (2002). Melatonin potentiates 5-HT(1A) receptor activation in rat hypothalamus and results in hypothermia. J Pineal Res.

[56] de Fronzo RA, Hendler R, Simonson D (1982). Insulin resistance is a prominent feature of insulin-dependent diabetes. Diabetes.

[57] Anhe GF, Caperuto LC, Pereira-Da-Silva M, Souza LC, Hirata AE, Velloso LA (2004). In vivo activation of insulin receptor tyrosine kinase by melatonin in the rat hypothalamus. J Neurochem.

[58] Zanquetta MM, Seraphim PM, Sumida DH, Cipolla-Neto J, Machado UF (2003). Calorie restriction reduces pinealectomy-induced insulin resistance by improving GLUT4 gene expression and its translocation to the plasma membrane. J Pineal Res.

