# Supplementary figures and images for: Streptozotocin-induced diabetes disrupts the body temperature daily rhythm in rats
Source: Diabetol Metab Syndr. 2015 Apr 29;7:39. doi: 10.1186/s13098-015-0035-2 (PMC4424512; doi:10.1186/s13098-015-0035-2)

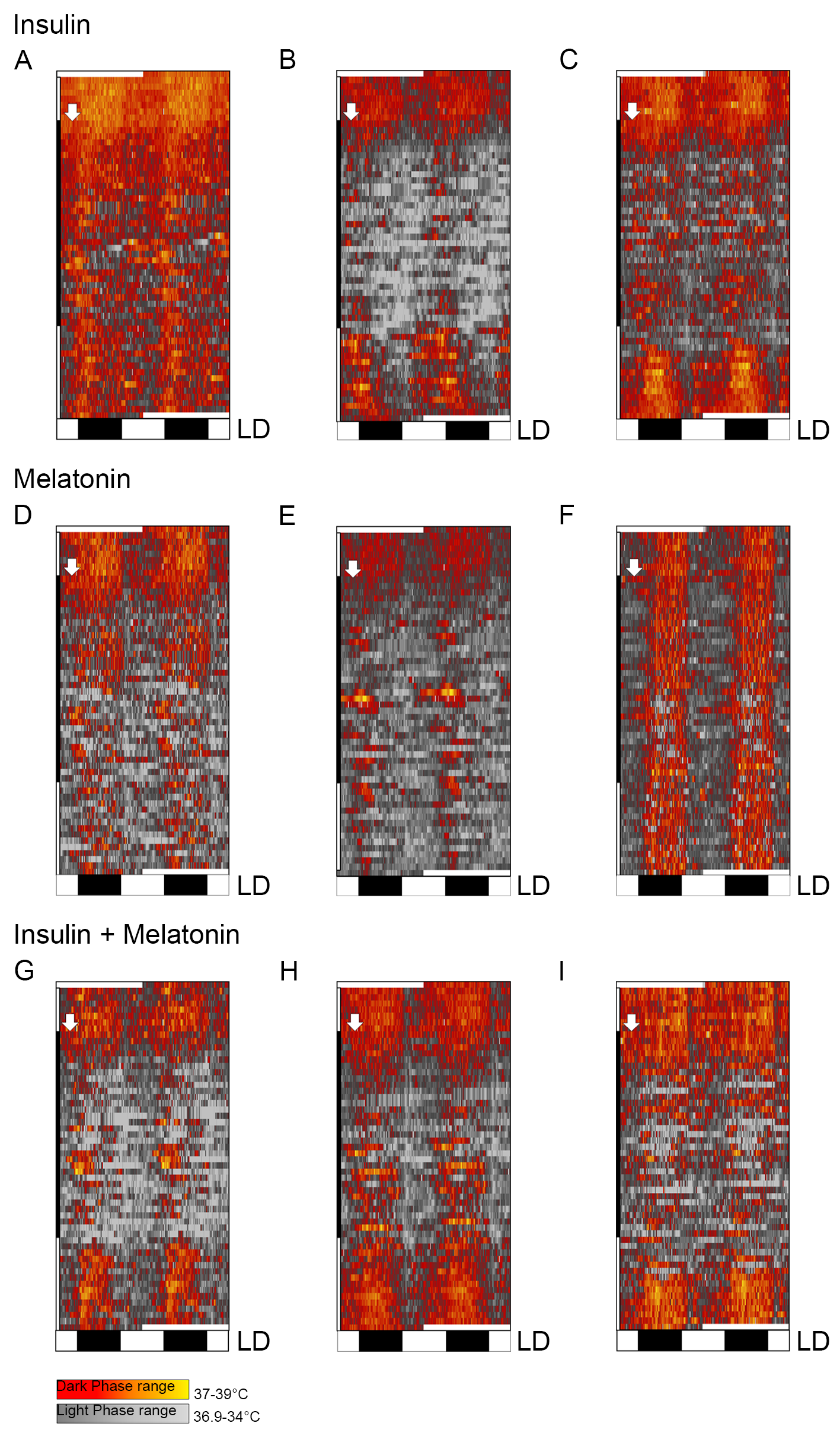

Supplement: Additional file 1: — Representative 48 h individual body temperature profiles in Late-INS (A), Late-MEL (B), and Late-INS + MEL (C) animals. [file 13098_2015_35_MOESM1_ESM.tiff]

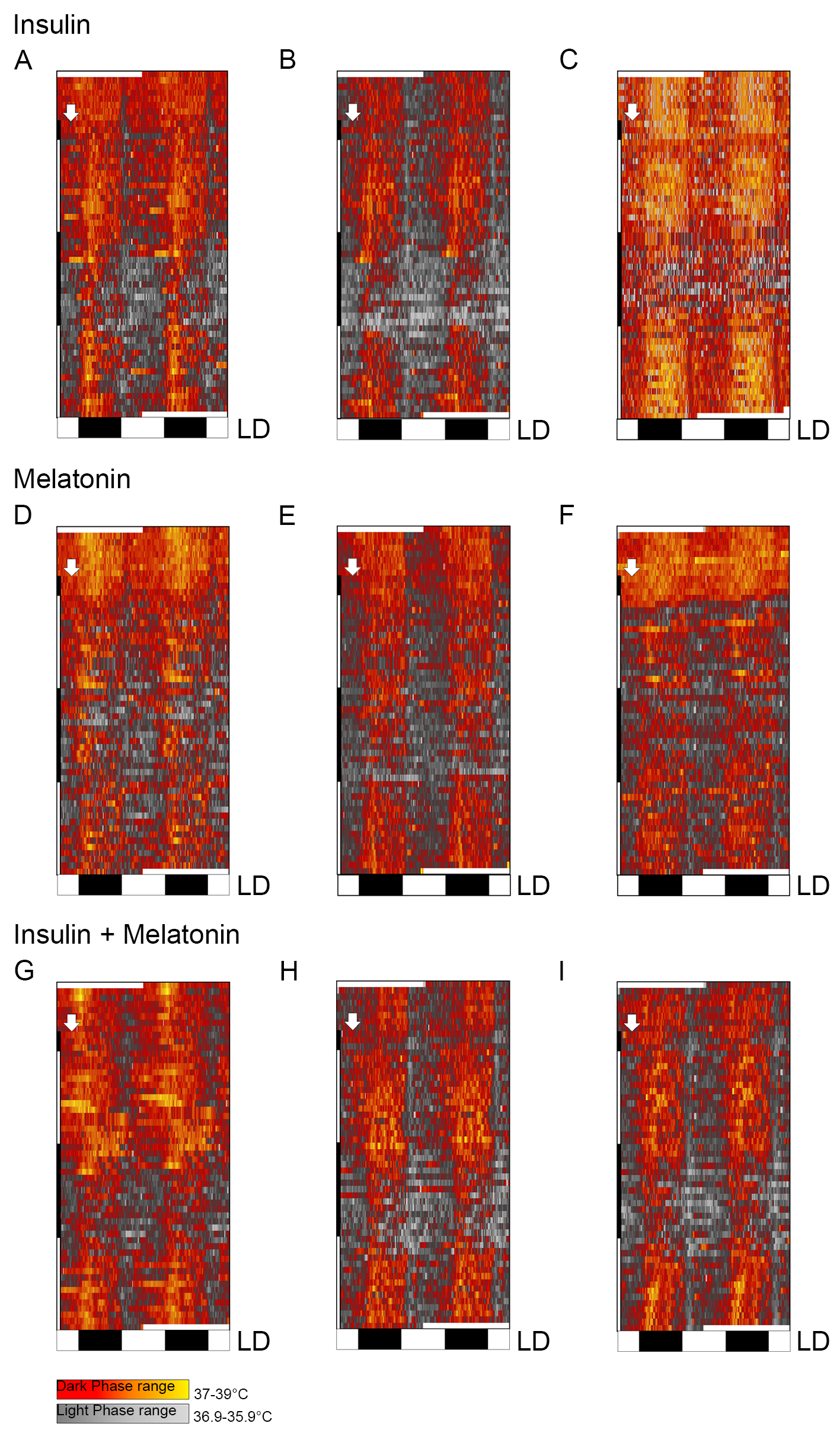

Supplement: Additional file 2: — Representative 48 h individual body temperature profiles in Early-INS (A), Early-MEL (B) and Early-INS + MEL (C) animals. [file 13098_2015_35_MOESM2_ESM.tiff]

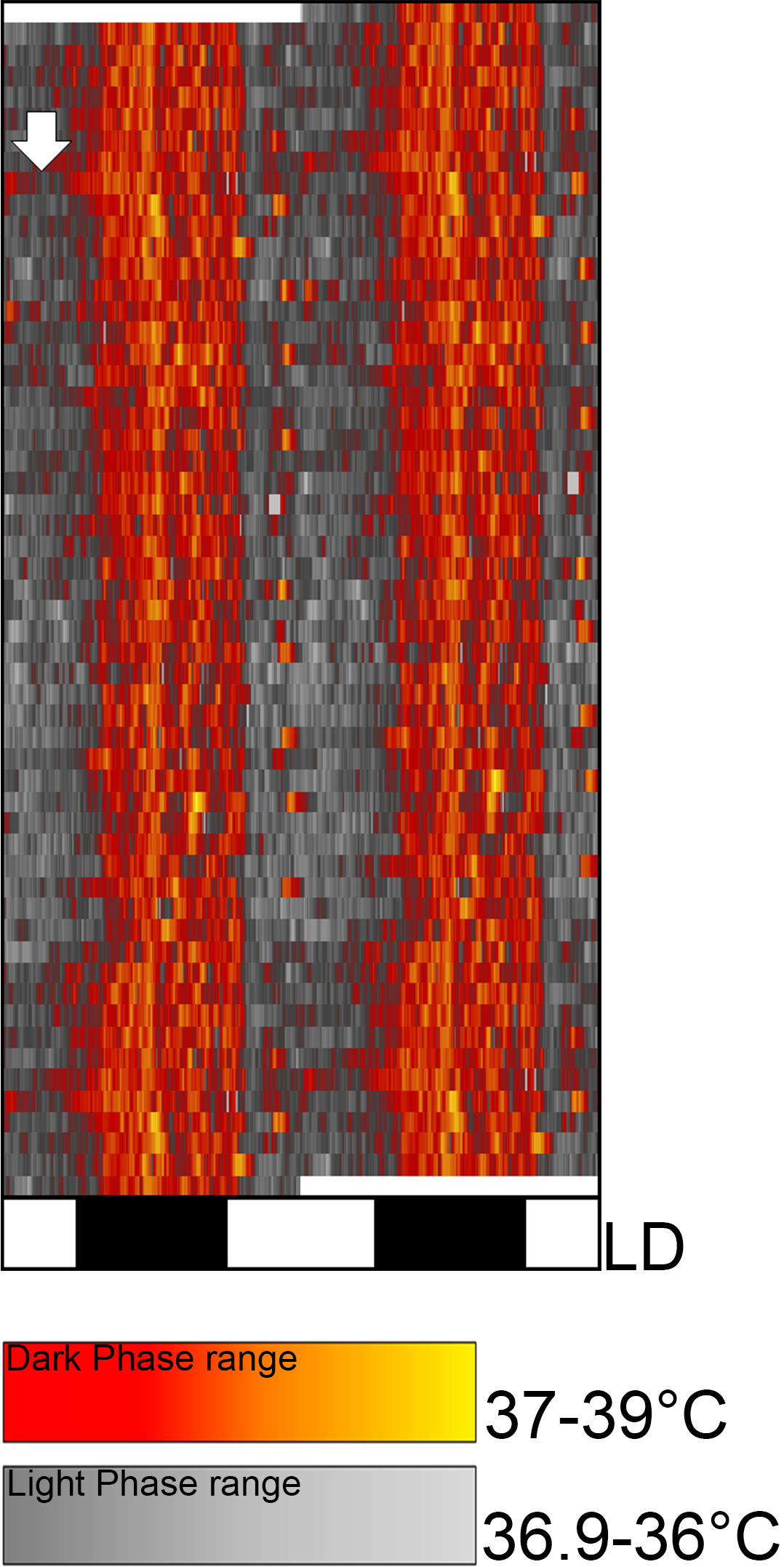

Supplement: Additional file 3: — Representative 48 h individual body temperature profiles in Control animals. Citrate buffer has no effect on body temperature profiles. [file 13098_2015_35_MOESM3_ESM.tiff]

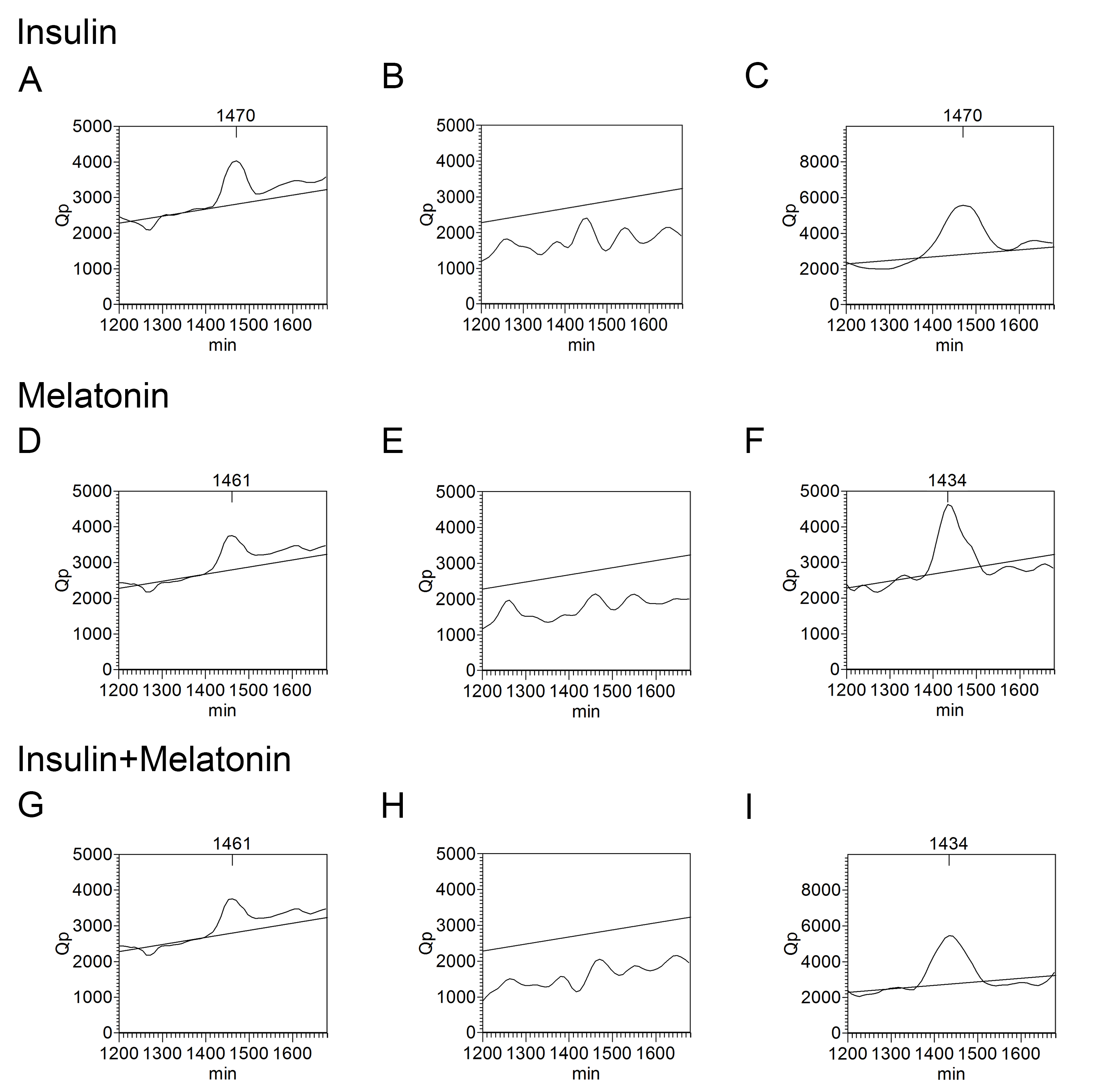

Supplement: Additional file 4: — Representative periodograms for Late-INS (A-C), Late-MEL (D-F) and Late-INS + MEL (G-I) animals indicating the predominant period of the control (A, D, G), diabetic (B, E, H) and treated phases (C, F, I). The threshold line indicates when a determined period was statistically predominant in the temporal series. [file 13098_2015_35_MOESM4_ESM.tiff]

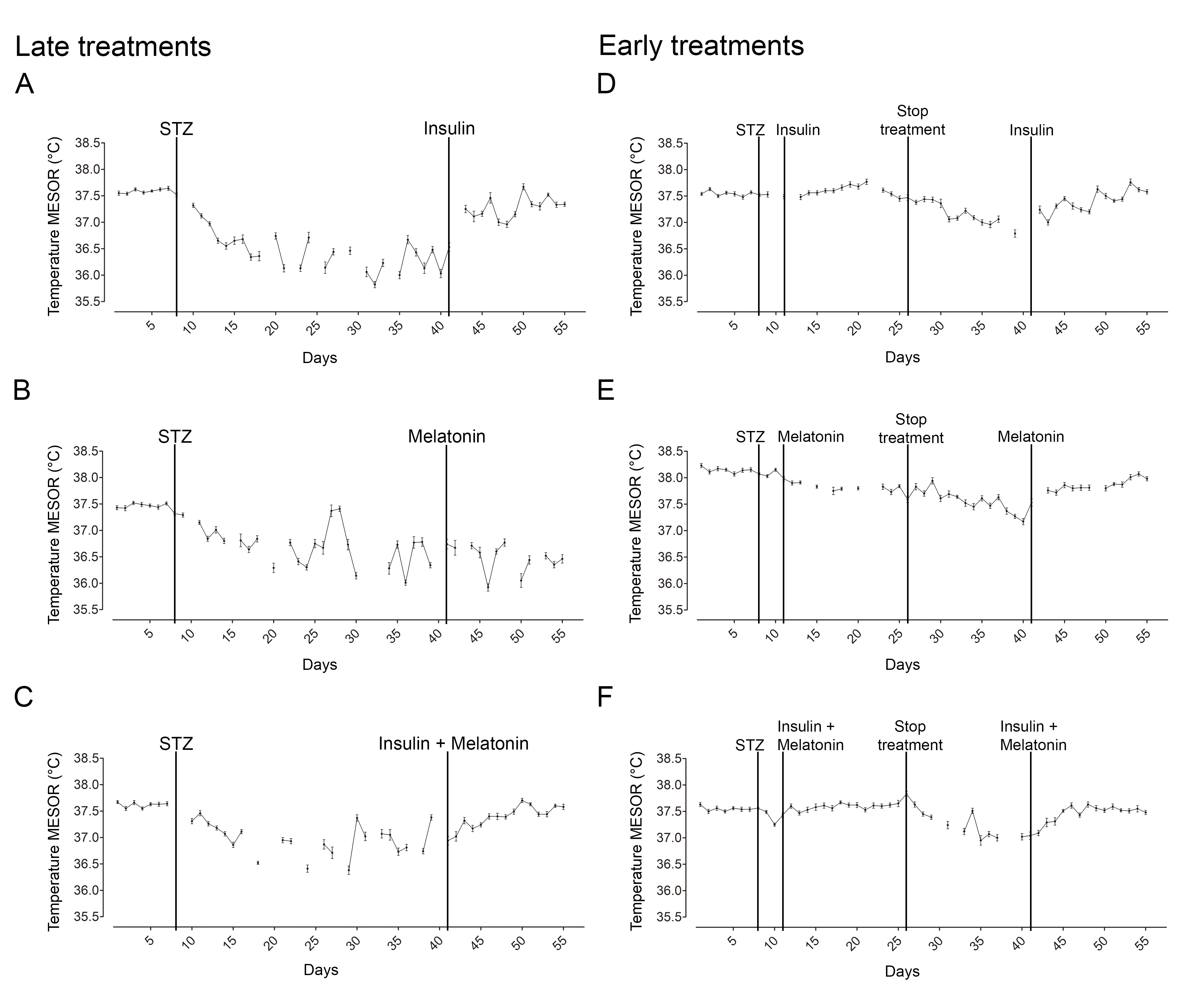

Supplement: Additional file 5: — Temperature MESOR resulting from day-to-day analyses of BT averaged in 1 h bins in late treatments (A-C) and early treatments (D-F) with insulin (A, D), melatonin (B, E) and insulin plus melatonin (C, F). Only days that showed 24 h oscillations were considered. [file 13098_2015_35_MOESM5_ESM.tiff]

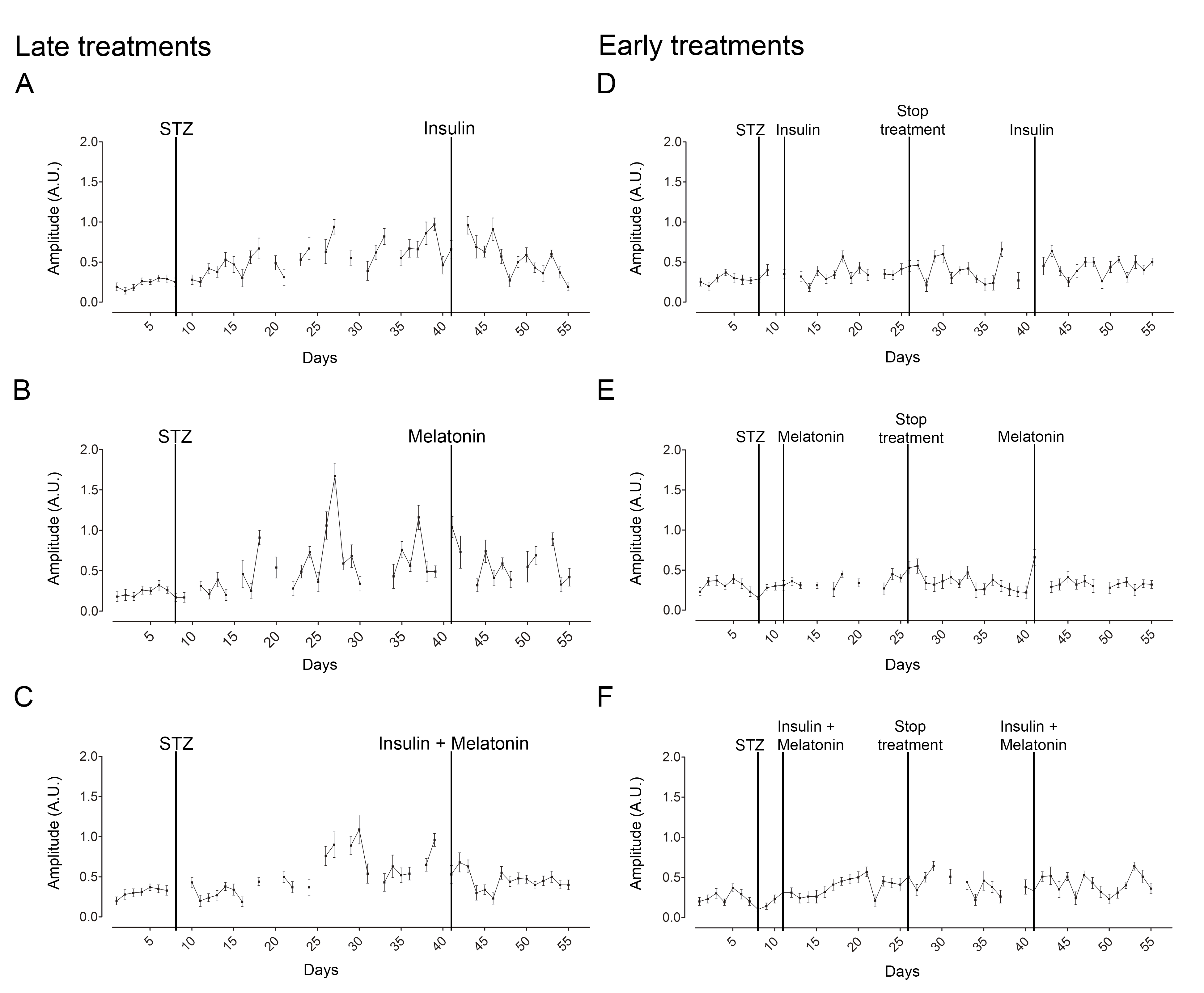

Supplement: Additional file 6: — Amplitude of the BT rhythm resulting from day-to-day analyses averaged in 1 h bins in late treatments (A-C) and early treatments (D-F) with insulin (A, D), melatonin (B, E) and insulin plus melatonin (C, F). Only days that showed 24 h oscillations were considered. [file 13098_2015_35_MOESM6_ESM.tiff]

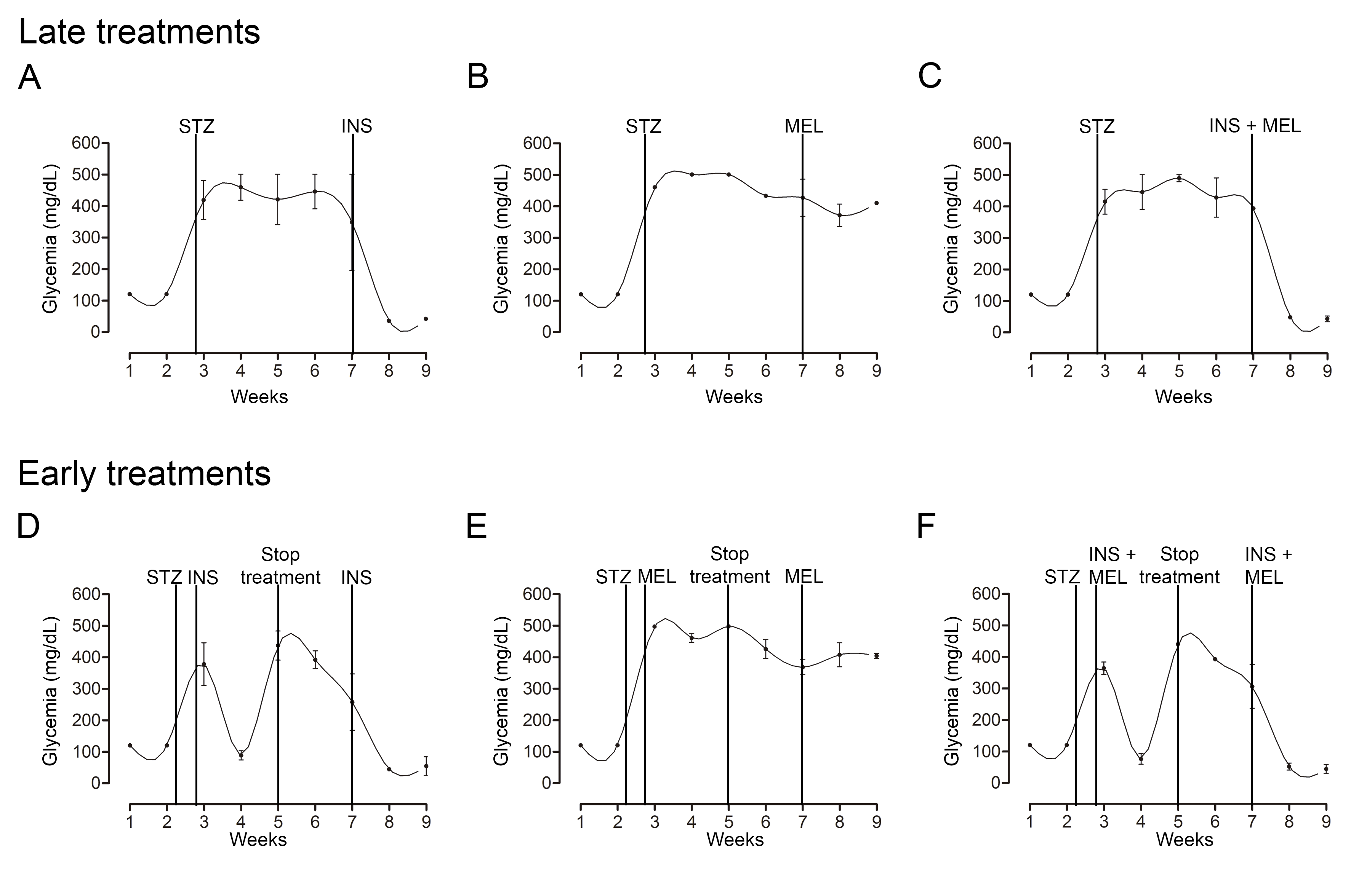

Supplement: Additional file 7: — Variations in glycemia averaged per week in diabetic animals treated with insulin (A, D), melatonin (B, E) or insulin plus melatonin (C, F). Upper row shows late treatments and bottom row, early treatments. Mean ± SEM. [file 13098_2015_35_MOESM7_ESM.tiff]

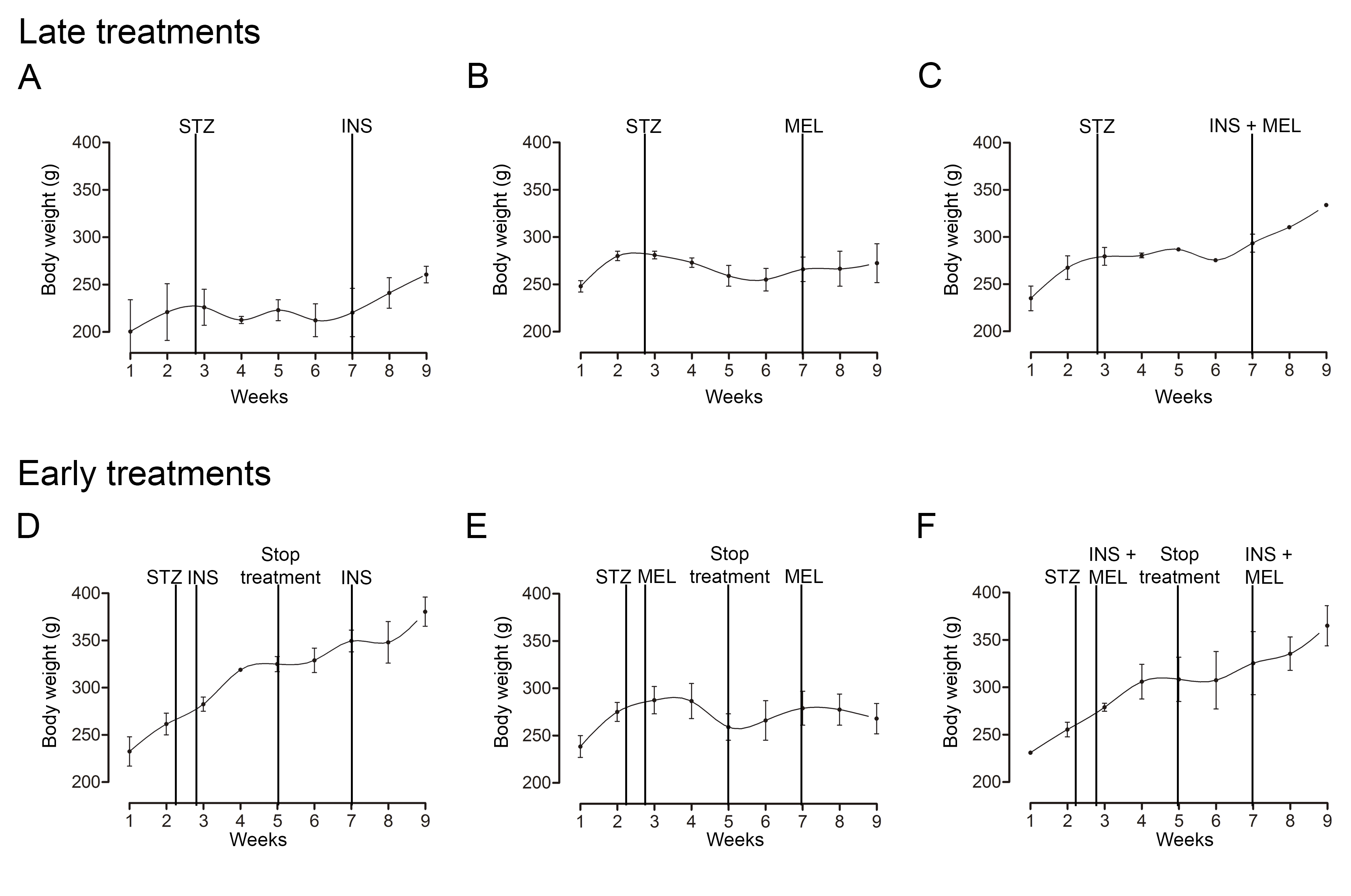

Supplement: Additional file 8: — Body weight of diabetic animals averaged per week treated with insulin (A, D), melatonin (B, E) and insulin plus melatonin (C, F). Upper row shows late treatments and bottom row, early treatments. Mean ± SEM. [file 13098_2015_35_MOESM8_ESM.tiff]

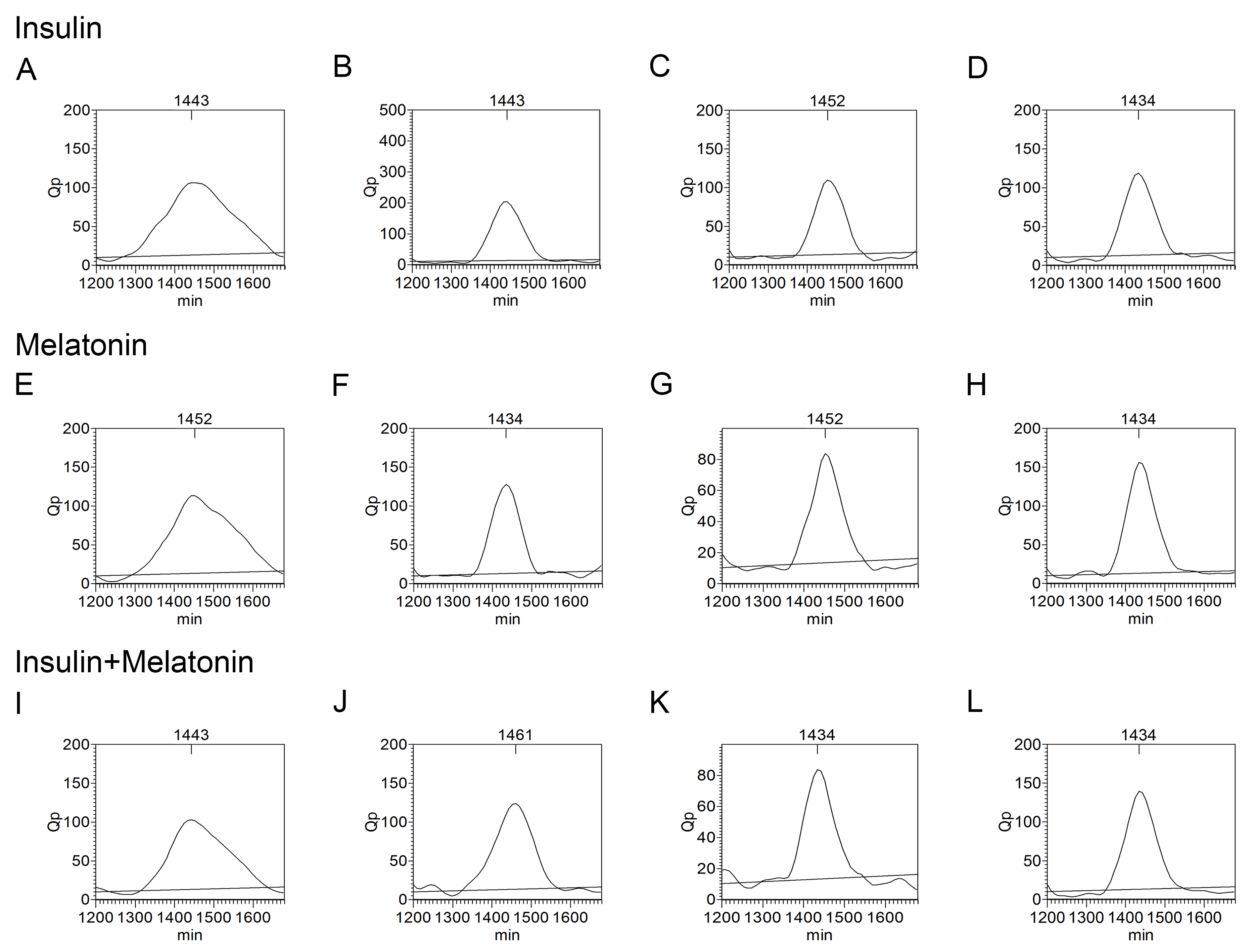

Supplement: Additional file 9: — Representative periodograms for Early-INS (A-D), Early-MEL (E-H) and Early-INS + MEL (I-L) animals indicating the predominant period of the control (A, E, I), early treatment (B, F, J), off-treatment (C, G, K) and restituting treatment phases (D, H, L). The threshold line indicates when a determined period was statistically predominant in the series. [file 13098_2015_35_MOESM9_ESM.tiff]
